# Engineered CHO cells as a novel AAV production platform for gene therapy delivery

**DOI:** 10.1038/s41598-023-46298-3

**Published:** 2023-11-06

**Authors:** Abdou Nagy, Lina Chakrabarti, James Kurasawa, Sri Hari Raju Mulagapati, Paul Devine, Jamy Therres, Zhongying Chen, Albert E. Schmelzer

**Affiliations:** 1 Cell Culture and Fermentation Sciences, Biopharmaceutical Development, BioPharmaceuticals R&D, AstraZeneca, One MedImmune Way, Gaithersburg, MD 20878 USA; 2 Biologics Engineering, R&D, AstraZeneca, One MedImmune Way, Gaithersburg, MD 20878 USA; 3 Analytical Science, Biopharmaceutical Development, Biopharma R&D, AstraZeneca, One MedImmune Way, Gaithersburg, MD 20878 USA; 4 Analytical Science, Biopharmaceutical Development, Biopharma R&D, AstraZeneca, Milstein Building, Granta Park, Cambridge, CB216GH UK; 5 Clinical Pharmacology and Safety Sciences, AstraZeneca, One MedImmune Way, Gaithersburg, MD 20878 USA

**Keywords:** Biotechnology, Drug discovery

## Abstract

The Herpes simplex virus (HSV)-based platform for production of recombinant adeno-associated viral vectors (rAAVs) yields higher titers and increased percentage of full capsids when compared to the triple transient transfection (TTT) method. However, this platform currently faces two major challenges. The first challenge is the reliance on commercial media, sometimes supplemented with serum, leading to costly manufacturing and a high risk for introduction of adventitious agents. The second challenge is that the production of HSV-1 relies on adherent complementing Vero cells (V27), making it difficult to scale up. We engineered serum-free-adapted CHO cells expressing key HSV-1 entry receptors, HVEM and/or Nectin-1 to address the first challenge. Using high-throughput cloning methods, we successfully selected a HVEM receptor-expressing clone (CHO–HV–C1) that yields 1.62 × 10^9^, 2.51 × 10^9^, and 4.07 × 10^9^ viral genome copies/mL with rAAV6.2-GFP, rAAV8-GFP, and rAAV9-GFP vectors respectively, within 24 h post rHSV-1 co-infection. Moreover, CHO–HV–C1-derived rAAVs had comparable in vitro transduction, infectivity, and biodistribution titers to those produced by TTT. The second challenge was addressed via engineering CHO–HV–C1 cells to express HSV-1 CP27. These cells successfully produced rHSV-1 vectors, but with significantly lower titers than V27 cells. Taken together, the CHO/HSV system provides a novel, scalable, reduced cost, serum-free AAV manufacturing platform.

## Introduction

Recombinant adeno-associated viral vectors (rAAVs) are the leading platform for gene therapy, with four licensed products approved by the end of 2022^1,2^. The rAAVs have several advantages as gene delivery vectors, including their ability to transduce a variety of proliferating and non-proliferating cells, accommodate cell/tissue-specific promoters, and elicit diminished immune response in comparison to other viral vectors^3^. Adeno-associated virus (AAV) is a small, nonenveloped virus in the genus *Dependovirus* within the family *Parvoviridae*^4,5^. The AAV 4.6-kb single-stranded DNA genome contains two viral genes, *rep* and *cap*. These genes can be replaced with a cassette expressing a therapeutic transgene along with the necessary *rep* and *cap* genes provided in *trans*^6^. AAV capsids are icosahedral and assembled from 60 viral protein (VP) monomers, with approximately 5 copies of VP1, 5 copies of VP2, and 50 copies of VP3^7^.

Currently, there are several different cell culture expression platforms for production of rAAVs: viral helper platforms, including infection of *Spodoptera frugiperda* (Sf9) insect cells with live baculovirus expression vector (BEV) and adenoviral infection of mammalian cells^8,9^; stable packaging cell lines, which express the *rep* and the *cap* genes of a desired rAAV serotype; stable proviral producer cell lines that stably express *rep*, *cap* and the transgene; and triple plasmid transient transfection (TTT)^10–12^. The triple transient transfection platform is the most common method for rAAV production, which uses three plasmids: one encoding the gene of interest (GOI) flanked by the AAV inverted terminal repeats (ITRs), a second encoding the *rep* and *cap* genes of AAV, and the third encoding the adenovirus helper function genes. This three-plasmid system can be simplified to a co-transfection by including the helper genes in the *rep*–*cap* plasmid^11,13–15^; however, scale-up remains a challenge. The BEV/Sf9 system is the second most common AAV production platform with several advantages, including ease of scaling-up and rAAV production with reduced encapsidation of contaminating DNA^16^. A recent study reported that rAAV capsids produced in either HEK293 cells using TTT or in BEV/Sf9 cells had different post-translational modifications (PTMs), such as glycosylation, phosphorylation, methylation, and acetylation. In addition, rAAVs produced in HEK293 cells using TTT were more potent in vitro than BEV/Sf9 vectors in various cell types and mouse tissue^17^.

Much like adenovirus and BEV, recombinant Herpes simplex virus-1 (rHSV-1) can also support rAAV replication as part of a helper virus system^18^. The minimum HSV-1 helper genes required for rAAV replication encode the HSV helicase primase complex (UL5, UL8, and UL52), the HSV DNA polymerase, and the HSV DNA-binding protein (UL29)^19^. Moreover, it has been reported that the rHSV co-infection method, which uses two HSV-1 vectors, one encoding the desired rAAV *rep*/*cap* serotype and the second encoding the GOI flanked by AAVs ITRs, generated in various cell lines multiple serotypes of rAAVs, with high potency and a very low DNase-resistant particle to infectious particle (DRP/ip) ratio^3^. Another advantage of the HSV helper system is its ability to replicate and provide helper functions in multiple mammalian cells, in contrast to the adenovirus helper-assisted rAAV production that requires human cell lines^20^. Additionally, it has been reported that there are quantitative, qualitative, and biological differences between rAAV9 produced using either the HSV infection system or TTT. The HSV production system produced higher rAAV9 vector titers, with a higher percentage of full capsids than TTT. The potency of rAAV9 from the two production systems was identical; however, HSV-mediated production led to a lower expression of both rep and cap but increased levels of AAV genome replication^21^.

Chinese hamster ovary (CHO) cells are predominantly used as expression hosts for production of recombinant monoclonal antibodies and other therapeutic proteins, which comprise the fastest growing segment of the biopharmaceutical industry^22^. Due to the regulatory acceptance and low manufacturing costs of CHO cells relative to human cells, using CHO cells for viral production is desirable. However, there are limited reports on the use of CHO cells for rAAV production, likely due to cellular restriction factors in these cells that can affect rAAV production and interfere with virus packaging, as has been reported for other viruses. For example, it has been shown that CHO cells did not support vaccinia virus replication at the stage of viral intermediate protein synthesis^23^. In addition, CHO cells are naturally resistant to productive HSV-1 infection, because they lack key receptors for HSV-1 entry and infection^24^. In this study, we engineered serum-free-adapted, suspension CHO cells to be permissive for HSV-1 entry and infection via expression of HVEM and/or Nectin-1 proteins. Following co-infection with rHSV-1 vectors encoding necessary elements for rAAV production, the final selected top CHO clone (CHO–HV–C1), which expresses only the HVEM receptor, produced satisfactory titers for multiple AAV serotypes using an in-house production medium. In addition, the rAAV-derived from CHO–HV–C1 showed comparable in vitro infectivity and biodistribution to rAAV produced by the TTT system.

## Results

### Generation of stable CHO pools

**Figure 1:**
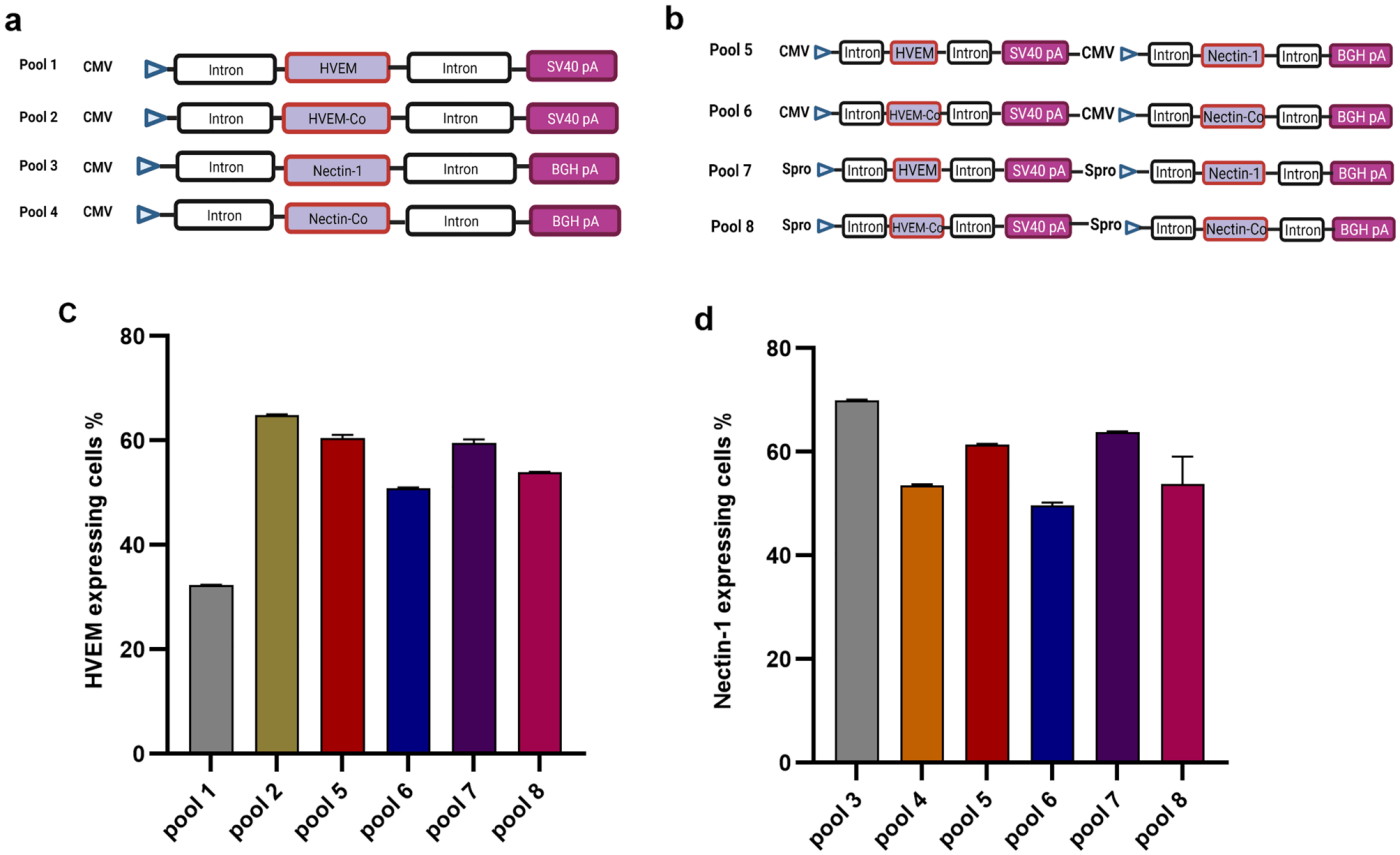
Plasmids constructs used for stable transfection and generation of stable pools. (**a**) Four individual cassettes: Pool 1 (CMV-HVEM) expressing HVEM; Pool 2 (CMV-HVEM-CO) expressing codon-optimized HVEM; Pool 3 (CMV-Nectin-1) expressing Nectin-1; and Pool 4 (CMV-Nectin-1-CO) expressing codon-optimized Nectin-1. The CMV promoter drives expression of all cassettes. (**b**) Four dual cassettes: Pool 5 (CMV-HVEM-Nectin-1) expressing both HVEM and Nectin-1; Pool 6 (CMV-HVEM-Nectin-1-CO) expressing codon-optimized HVEM and Nectin-1; Pool 7 (Spro-HVEM-Nectin-1) expressing HVEM and Nectin-1; and Pool 8 (Spro-HV-NE-CO) expressing codon-optimized HVEM and Nectin-1. The CMV promoter drives expression of gene cassettes in pools 5 and 6 while synthetic (Spro) promoter drives expression of gene cassettes in pool 7 and 8. (**c**) Percentage of HVEM expressing cells from pools: 1, 2, 5, 6, 7 and 8. (**d**) Percentage of Nectin-1 expressing cells from pools: 3, 4, 5, 6, 7 and 8. Figures (**a**) and (**b**) were created with BioRender.com.

Eight different vectors using an in-house plasmid backbone expressing glutamine synthetase (GS) were constructed: these included: (1) two vectors encoding either codon-optimized or non-codon-optimized HVEM flanked by the CMV promoter and the SV40 polyA; (2) two vectors encoding either codon-optimized or non-codon-optimized Nectin-1 flanked by CMV promoter and BGH polyA; (3) two vectors encoding either codon-optimized or non-codon-optimized HVEM and Nectin-1 ORFs flanked by CMV promoter, as well as SV40 polyA and BGH polyA, respectively; and (4) two vectors encoding either codon-optimized or non-codon-optimized HVEM and Nectin-1 ORFs flanked by a synthetic promoter (Spro), SV40 polyA and BGH polyA, respectively (Fig. 1a,b**)**.

After ten days of methionine sulfoximine (MSX) selection, an aliquot of each recovered stable cell pool (1 × 10^6^ viable cells/pool) was tested for HVEM and/or Nectin-1 receptor expression using flow cytometry. Pools 1 (CMV-HVEM), 2 (CMV-HVEM-Co), 5 (CMV-HVEM-Nectin-1), 6 (CMV-HVEM-Co-Nectin-1-Co), 7 (Spro-HVEM-Nectin-1), and 8 (Spro-HVEM-Co-Nectin-1-Co) showed 32.4%, 65%, 60.9%, 51%, 60%, 53.8% HVEM expression, respectively (Fig. 1c). Pools 3 (CMV-Nectin-1), 4 (CMV-Nectin-1-Co), 5 (CMV-HVEM-Nectin-1), 6 (CMV-HVEM-Co-Nectin-1-Co), 7 (Spro-HVEM-Nectin-1) and 8 (Spro-HVEM-Co-Nectin-1-Co) showed 69.75%, 53.45%, 61.3%, 49.3%, 63.7% and 53.8% Nectin-1 expression, respectively (Fig. 1d). Interestingly, pools 5, 6, 7 and 8 showed remarkable dual expression of HVEM and Nectin-1, indicating CMV and synthetic promoters are comparable in driving high level of expression of HVEM and Nectin-1 proteins. Moreover, no significant differences in HVEM and Nectin-1 receptor expression using either non-codon-optimized and/or codon-optimized protein versions were observed.

### rHSV-1 infection and rAAV9-GFP production in stable CHO pools

**Figure 2:**
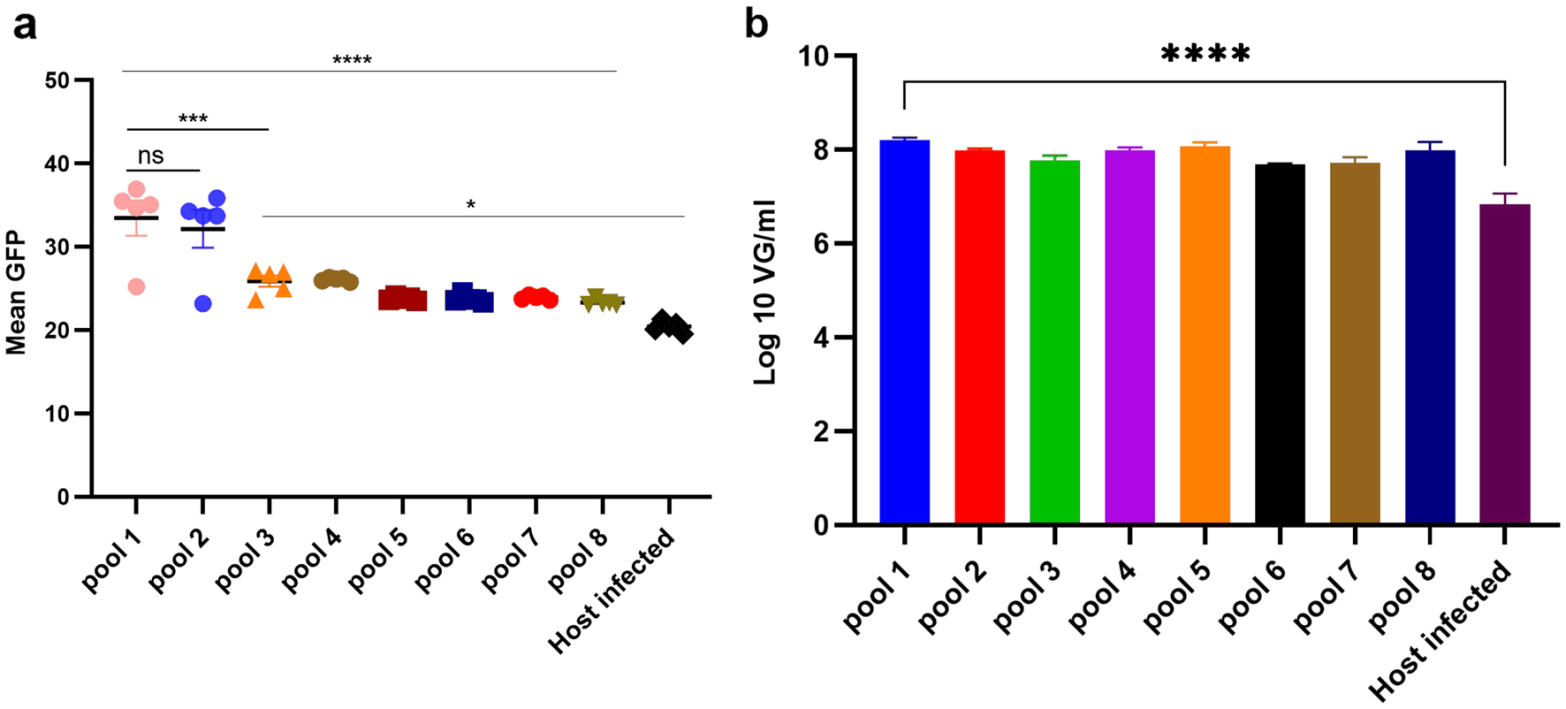
GFP expression and rAAV9-GFP production from stable CHO cell pools. (**a**) Mean GFP expression from rHSV1-GFP infected stable cell pools (MOI = 10) from total five time points post-infection. Data were analyzed with one-way ANOVA and expressed as mean with SEM. The ns means non-significant,****p-value < 0.0001, ***p = 0.0004, *p = 0.016. (**b**) qPCR rAAV9-GFP titers (vg/mL) from supernatant of infected stable cell pools 24 hpi using an MOI of 1:1 of rHSV1-GFP to rHSV-AAV9 vectors. ****p-value < 0.0001. All samples were tested in duplicate, and data were tested with one-way ANOVA, and data presented as mean ± standard deviation (SD).

With the HVEM and Nectin-1 receptors stably expressed in CHO pools, it was critical to determine which of the eight constructs provided the best rHSV-1 infection and subsequent rAAV expression after coinfection with rHSV-1 vectors. Each of the stable pools generated above were infected with rHSV1-GFP at a multiplicity of infection (MOI) of 10 plaque forming units (PFU) per cell, with the wild-type CHO cells infected as a negative control. GFP expression data were collected from all the infected stable cell pools (n = 8) every 12 h post-infection (hpi) up to 60 h. All eight infected cell pools showed significant mean GFP expression starting at 12 hpi, when compared to the infected wild-type CHO cells (Fig. 2a). The highest level of GFP expression was observed at 12 hpi in the HVEM-only expressing pools (pools 1 and 2), with a *p*-value < 0.0001 when compared to the infected wild-type CHO cells. The next highest level of GFP expression came from Nectin-1-only expressing pools (pools 3 and 4), with a *p*-value < 0.016 when compared to the infected wild-type CHO cells (Fig. 2a).

To determine rAAV production from the stable pools, cell supernatant was collected a day after co-infection with rHSV-1 encoding AAV2 rep and AAV9 cap proteins, as well as rHSV-1 encoding GFP flanked by AAV2 ITRs, and rAAV9-GFP vector was titrated using qPCR. Pool 1 showed the highest rAAV9-GFP titer with an average of 1.58 × 10^8^ vg/mL, compared to pools 2 through 8 that ranged between an average of 5.13 × 10^7^ and 1.17 × 10^8^ vg/mL, although this value was not significantly higher than the titers produced by the other pools. All stable pools produced more rAAV9-GFP than wild-type CHO cells (6.92 × 10^6^ vg/mL, *p* < 0.0001 (Fig. 2b). These data indicate pool 1 outperformed all other pools for rHSV1-GFP infection, GFP expression, and rAAV-GFP production. Therefore, this pool was selected for a single-cell cloning.

### Selection of top CHO clones for HSV-1 infection and AAV production

**Figure 3:**
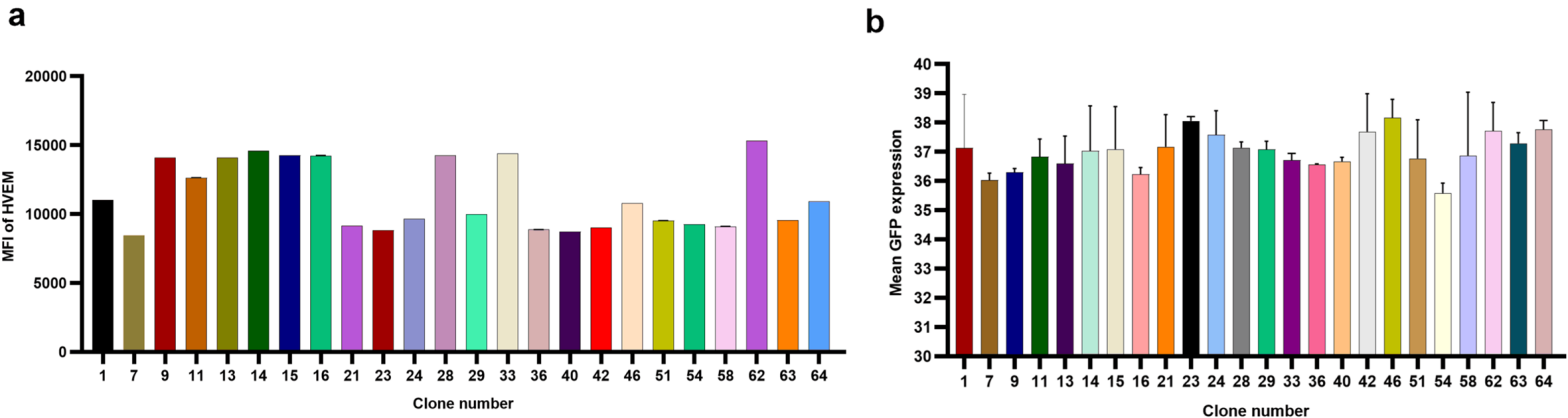
Testing CHO-HVEM expressing clones for HVEM surface expression and rHSV-1-GFP infection. (**a**) Mean Fluorescence Intensity of the top 24 CHO-HVEM expressing clones. (**b**) Mean GFP expression from the final 24 clones post rHSV-1 GFP infection using the IncuCyte. Data from the mean GFP expression were analyzed using the Kruskal–Wallis test with Dunn’s multiple comparisons. Data (n = 2 per sample) presented as mean ± SD.

High and medium HVEM-expressing CHO cell subpopulations were identified and selected by fluorescence activated cell sorting (FACS) gating strategy and single cells from each of the gated populations (Supplementary Fig. S1)were deposited individually into a well of a 384-well plate. Deposition of single cell per well was verified by imaging using Cellavista^25^. After two weeks, 64 clones with a satisfactory outgrowth were selected for further evaluation. The selected clones were scaled up using 96-deep well plates and passaged using in-house medium supplemented with MSX. After three passages, 24 out of the initial 64 clones showed good HVEM expression by flow cytometry (Fig. 3a). These clones were expanded and further tested for rHSV1-GFP vector infection. The mean GFP expression, determined at 24 and 48 hpi, showed no significant difference among the tested clones (Fig. 3b).

**Figure 4:**
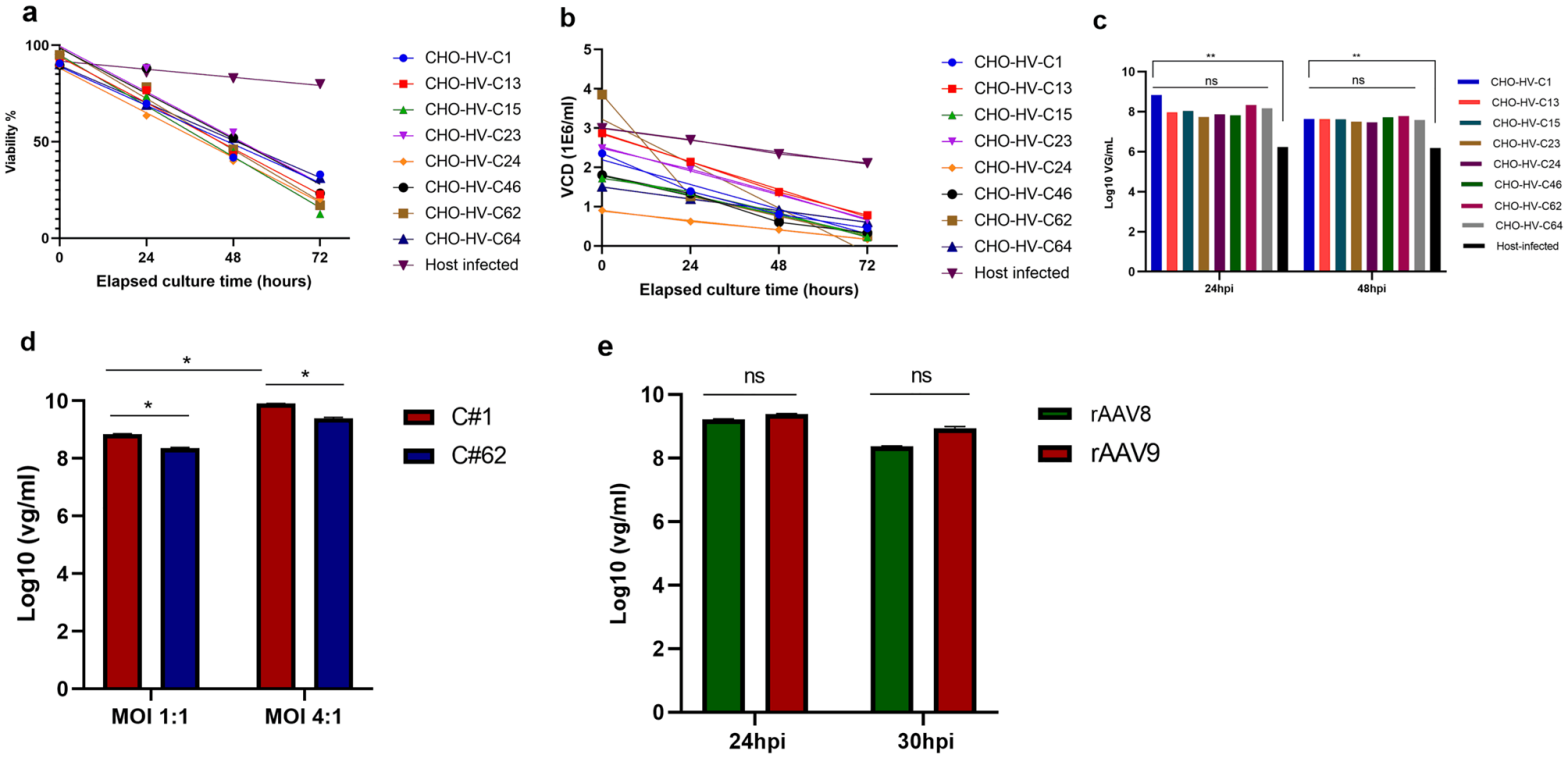
Evaluation of production of rAAV6.2-GFP in top eight CHO-HVEM clones. (**a**) Cell viability plots post-rHSV-1 vectors co-infection. (**b**) Viable cell density plots post-rHSV-1 vectors co-infection. (**c**) The rAAV6.2-GFP titers using an MOI of 1:1. Ns means non-significant, **p = 0.0015. (**d**) The rAAV6.2-GFP production in CHO–HV–C1 and CHO–HV–C62 clones using different MOIs. *p = 0.051. (**e**) Production of rAAV8-GFP and rAAV9-GFP in CHO–HV–C1 cells at 24 and 30 hpi.

Eight clones originated from CMV-HVEM-SV40 polyA (C1, C13, C15, C23, C24, C46, C62, and C64) showed the highest mean GFP expression post rHSV1-GFP infection. These eight clones were selected to further assess their capability to produce rAAV6.2-GFP via co-infection at an MOI of 1:1 PFU/cell of rHSV-AAV2 rep/cap6.2 and rHSV-GFP. Over the course of the co-infection, the viability of these eight clones decreased rapidly, compared to only a slight decrease (13%) of the infected wild-type CHO cells, when incubated at 37 °C (Fig. 4a). Likewise, the viable cell density (VCD) was also reduced for all co-infected clones compared to the minor VCD drop observed (by 0.3 × 10^6^ cells/mL) for the infected wild-type CHO cells (Fig. 4b). It seems likely that the AAV rep protein exerts a cytotoxic effect on the infected engineered CHO cells, as previously reported for other cell lines such as HL-60 cells^26^. The cytotoxic effect is not observed in infected wild-type CHO host cells due to the degradation of rHSV-1 vectors after cellular entry. We found clone #1 (CHO–HV–C1) produced the highest rAAV6.2-GFP vector titer per mL of cell lysate (~ 6.76 × 10^8^ vg/mL from 1 × 10^6^ cells) at 24 hpi, compared to other clones that produced between 5.50 × 10^7^ and 2.19 × 10^8^ vg/mL. Although the rAAV6.2-GFP vector titer produced in the CHO–HV–C1 clone was not statistically different from titers produced by the other clones (*p* = *0.89)*, all clones had significantly higher titers than the co-infected wild-type CHO host cells, which produced 1.74 × 10^6^ vg/mL at 24 hpi (*p* = 0.0015). It is also worth noting that for all eight co-infected clones, the rAAV6.2-GFP titers decreased at 48 hpi. Interestingly, although CHO–HV–C1 is a high HVEM expressing clone and CHO–HV–C62 is a medium HVEM expressing clone, both produced the highest titers of rAAV6.2-GFP vector (Fig. 4c). Therefore, further investigation is necessary to determine if HVEM expression levels have any impact on rAAV production.

In a subsequent experiment, we tested both top clones (CHO–HV–C1 and CHO–HV–C62) for rAAV6.2-GFP vector production using MOIs of 2:1, 3:1, and 4:1 PFU/cell of rHSV-AAV6.2 and rHSV1-GFP, respectively. Interestingly, MOIs of 2:1 and 3:1 did not show significant improvement in rAAV6.2-GFP titers per 1 × 10^6^ cells lysate at 24 hpi, compared to that obtained using MOI 1:1 (data not shown). On the other hand, MOI 4:1 significantly improved rAAV6.2-GFP titers produced in CHO–HV–C1 and CHO–HV–C62 clones (*p* = 0.051). In addition, CHO–HV–C1 outperformed CHO–HV–C62 for production of rAAV6.2-GFP vector at MOI 4:1, yielding 7.76 × 10^9^ and 2.34 × 10^9^ vg/mL, respectively, per 1 × 10^6^ cells lysate at 24 hpi (*p* = 0.026) (Fig. 4d). This indicates that the MOI of rHSV-1 has a major impact on the rAAV6.2-GFP yield in CHO cells.

As CHO–HV–C1 was the most productive clone for rAAV6.2-GFP production, we wanted to further explore its capacity to produce rAAV8 and rAAV9 expressing the GFP transgene. Using the same optimal infection parameters described in the previous experiment, average titers of 1.62 × 10^9^ and 2.51 × 10^9^ vg/mL per 1 × 10^6^ cells cell lysate for rAAV8-GFP and rAAV9-GFP vectors, respectively, were produced at 24 hpi. However, harvesting at 30 hpi resulted in decreased titers (8.36 and 9.55 × 10^8^ vg/mL for rAAV8-GFP and rAAV9-GFP vectors, respectively; Fig. 4e). These data indicate that the infection parameters described above are suitable for a variety of serotypes, with optimal titer achieved at 24 hpi. Similar to rAAV6.2-GFP vector production, the co-infected CHO–HV–C1 cells for the production of rAAV8-GFP and rAAV9-GFP vectors showed a sharp decrease in viability at 24 hpi, indicating that the effect on cell viability is not serotype-specific. We hypothesized that a sharp decline in the cell viability after rHSV-1 vector co-infection may affect the final rAAV titers. Thus, anticipating an extension of culture duration we evaluated the effect of a temperature switch from 37 to 33 °C at the time of co-infection with rHSV-1 AAV6.2 and rHSV-1-GFP at MOI 4:1. At 24 hpi, the co-infected cultures with a temperature switch showed improved cell viability over cultures incubated at 37 °C (Supplementary Fig. S2a), together with a slight improvement in rAAV6.2-GFP titers from cell lysate, as well as an increase in titer within the medium of approximately one log (Supplementary Fig. S2b). This finding indicates that post co-infection incubation temperature is an essential factor for cell viability and rAAV production in the CHO platform.

### Analytical characterization of CHO-derived rAAVs

**Figure 5:**
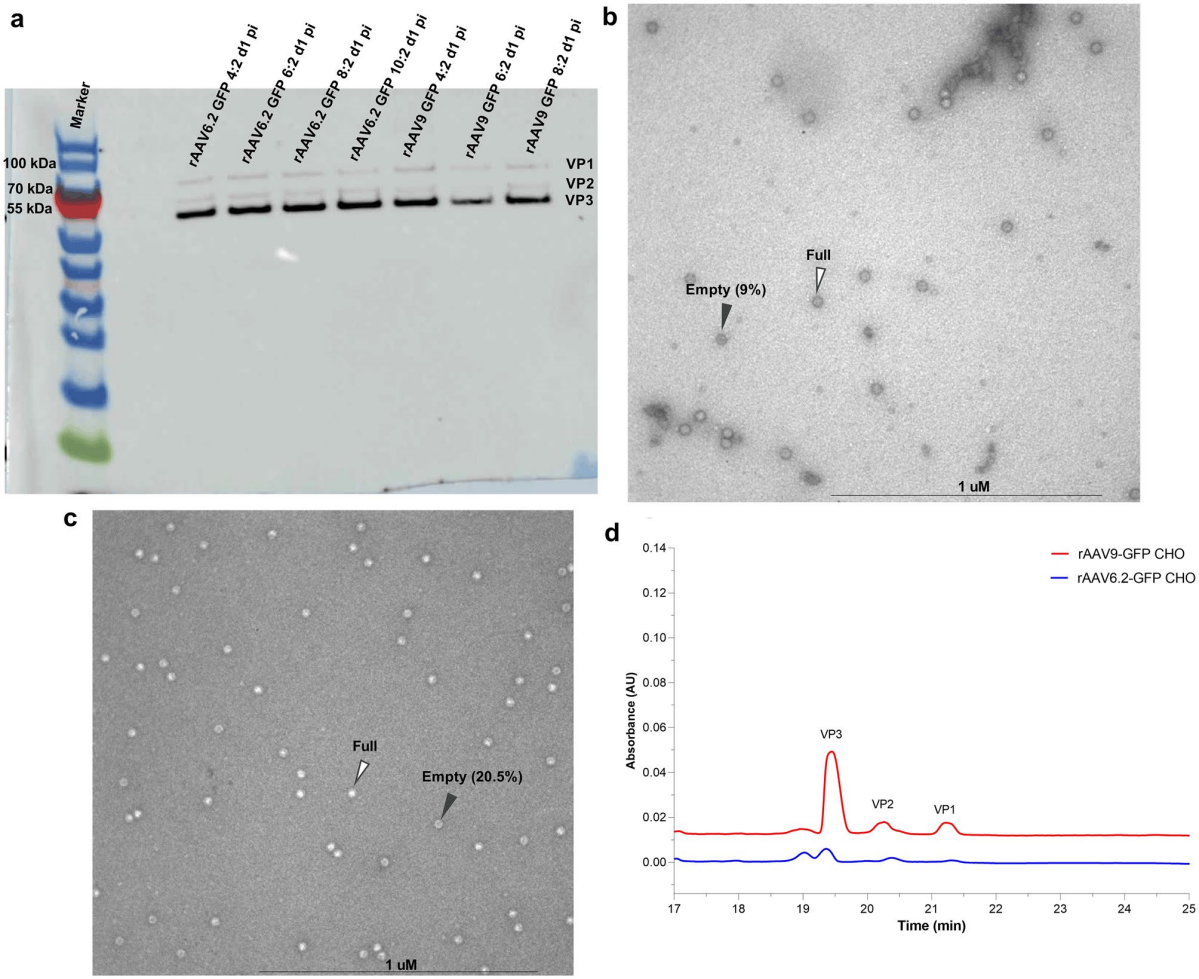
Analytical characterization of rAAVs produced in the CHO–HV–C1 clone. (**a**) Western blot of VP1, VP2 and VP3 capsid proteins from either purified rAAV6.2-GFP or rAAV9-GFP vectors produced with different MOIs in CHO–HV–C1 cells. (**b**) Mini transmission electron (Mini-TEM) micrograph of purified rAAV6.2-GFP. (**c**) Mini-TEM micrograph of purified rAAV9-GFP. White arrows indicate average full capsids, and black arrows indicate average non-full (empty) capsids. (**d**) The rAAV capsid ratio detection using CE-SDS method.

The PEG-Chloroform purification method has been reported as a fast, capsid-independent, and easy purification method for rAAV^27–29^. The entire bench-scale process of harvesting and purifying the rAAV vectors using PEG-Chloroform takes approximately one day (Supplementary Fig. S3). The final purified rAAV is stored at − 80 °C. In order to understand if VP expression ratios played a role in limiting rAAV production a higher MOI, purified rAAV were evaluated using Western blots. These purified rAAVs produced in CHO–HV–C1 clone using different MOIs of rHSV-1 showed comparable expression of VP1, VP2, and VP3 AAV capsid proteins (Fig. 5a). Moreover, examination of the purified rAAV6.2-GFP and rAAV9-GFP vectors produced in CHO cells using mini-TEM showed 91% and 79.5% full capsids, respectively (Fig. 5b,c), whereas the full capsid percentages for purified rAAV6.2-GFP and rAAV9-GFP vectors produced using TTT were 65.9% and 76.9%, respectively (data not shown) Analysis of the molar ratio of VP1:VP2:VP3 in the purified samples were consistent with those reported in the literature (Fig. 5d). These data show that rAAV vectors produced in our engineered CHO cells have good expression of capsid proteins with a high percentage of full AAV capsids.

In a subsequent experiment, purified rAAV6.2-GFP and rAAV9-GFP vectors were examined for any residual infectious HSV-1 vectors in the PEG-Chloroform purified materials by inoculating 10^10^ vg/mL of each vector on an HSV-1 complementing cell line (V27), with rHSV1-GFP vector (MOI 0.15 PFU/cell) as a positive control. No cytopathic effects appeared in wells inoculated with either purified rAAV6.2-GFP or rAAV9-GFP vectors up to 4 days post-infection. Conversely, the typical cytopathic effects, such as rounding of infected-cells and cell sheet detachment, appeared on day 2 post-infection in the well inoculated with rHSV1-GFP vector, with complete detachment of the cell sheet on day 3 post-infection (Supplementary Fig. S4). These data indicate that the in-house developed PEG-Chloroform purification method is efficient in inactivating rHSV-1 vectors as indicated by no detection of residual infectious rHSV-1 in the purified rAAV, thereby ensuring that competent HSV-1 are not carried over into the manufacturing process.

**Figure 6:**
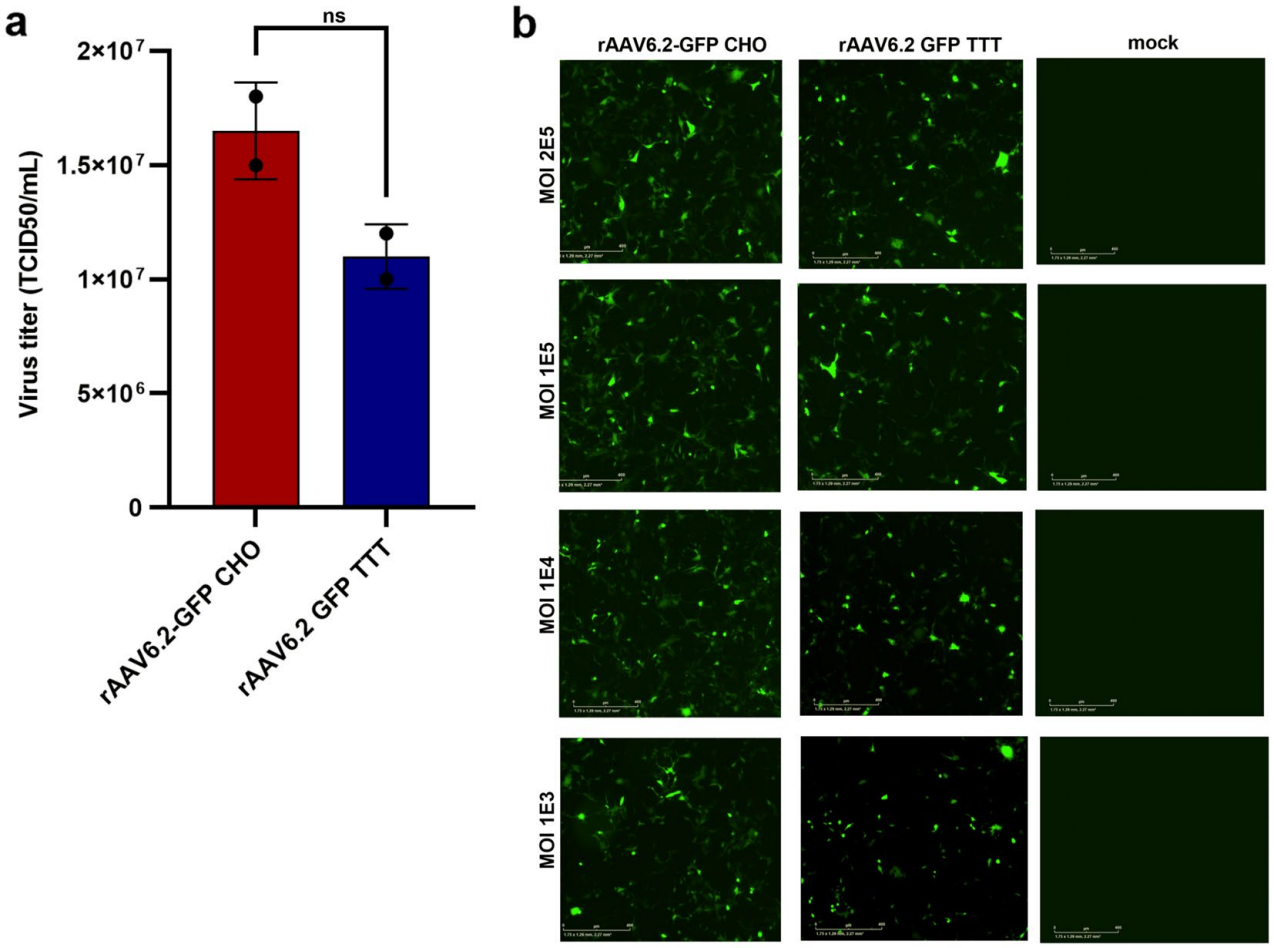
Infectivity of rAAVs produced in CHO–HV–C1 cells compared to HEK293. (**a**) Infectivity of purified rAAV6.2-GFP vector produced in either CHO–HV–C1 clone using rHSV-1 co-infection and/or HEK293 cells using triple transfection via TCID_50_. Data (n = 2 per sample) were analyzed with two-tailed Mann–Whitney test and expressed as mean ± SD. Individual biological replicates are shown for each group in the bar graph. (**b**) In vitro transduction of Ad293 cells with rAAV6.2-GFP produced in either CHO–HV–C1 cells using rHSV-1 co-infection or HEK293 cells using triple transfection. Mock infection of cells used PBS alone.

Transduction is the process by which AAV infects cells. This process includes several steps: binding and penetration of cells, intracellular trafficking, uncoating, and nuclear transport. By utilizing in vitro transduction, we can understand the ability of the produced AAV to carry out the aforementioned steps. Our aim was to compare the in vitro potency of CHO-derived rAAVs to rAAVs produced using the standard TTT method. For infectivity, GFP expression from infected wells was recorded on day 5 post-infection using the IncuCyte. The rAAV6.2-GFP vectors produced in the CHO–HV–C1 clone (rAAV6.2-GFP CHO) and the rAAV6.2-GFP produced in HEK293 cells (rAAV6.2-GFP TTT) showed comparable infectivity titers of 1.65 × 10^7^ and 1.1 × 10^7^ TCID_50_/mL, respectively (Fig. 6a).

To investigate the transduction efficiency of the two vector preparations mentioned above, Ad293 cells were infected with different MOI, ranging from 2 × 10^5^ down to 1 × 10^3^ vg/cell. The mean GFP expression was recorded on day 3 post-infection using the IncuCyte. The rAAV6.2–GFP–CHO and rAAV6.2–GFP–TTT showed potent transduction at all of the MOI tested (Fig. 6b). These data indicate that rAAV6.2–GFP-CHO has comparable in vitro infectivity and transduction activity to rAAV6.2–GFP–TTT.

### Biodistribution of CHO-derived AAVs

The biodistribution of rAAV6.2-GFP-CHO, in parallel with the rAAV6.2–GFP–TTT, was assessed to determine if the in vivo behavior mimicked the in vitro data. Fifteen 3-week-old mice were divided into three groups (n = 5 per group). The mice were inoculated with either rAAV6.2-GFP-CHO (G1), rAAV6.2–GFP–TTT (G2), or PBS (G3). All mice were inoculated with either 10^11^ vg rAAV or 100 µL of PBS intravenously in tail vein (TV), as per their grouping. A previous study showed that rAAV6 had high level of transduction in liver, skeletal muscle, kidney, lung, and heart in mouse post TV inoculation^30^. Therefore, these tissues were harvested from each inoculated animal three weeks post-inoculation.

**Figure 7:**
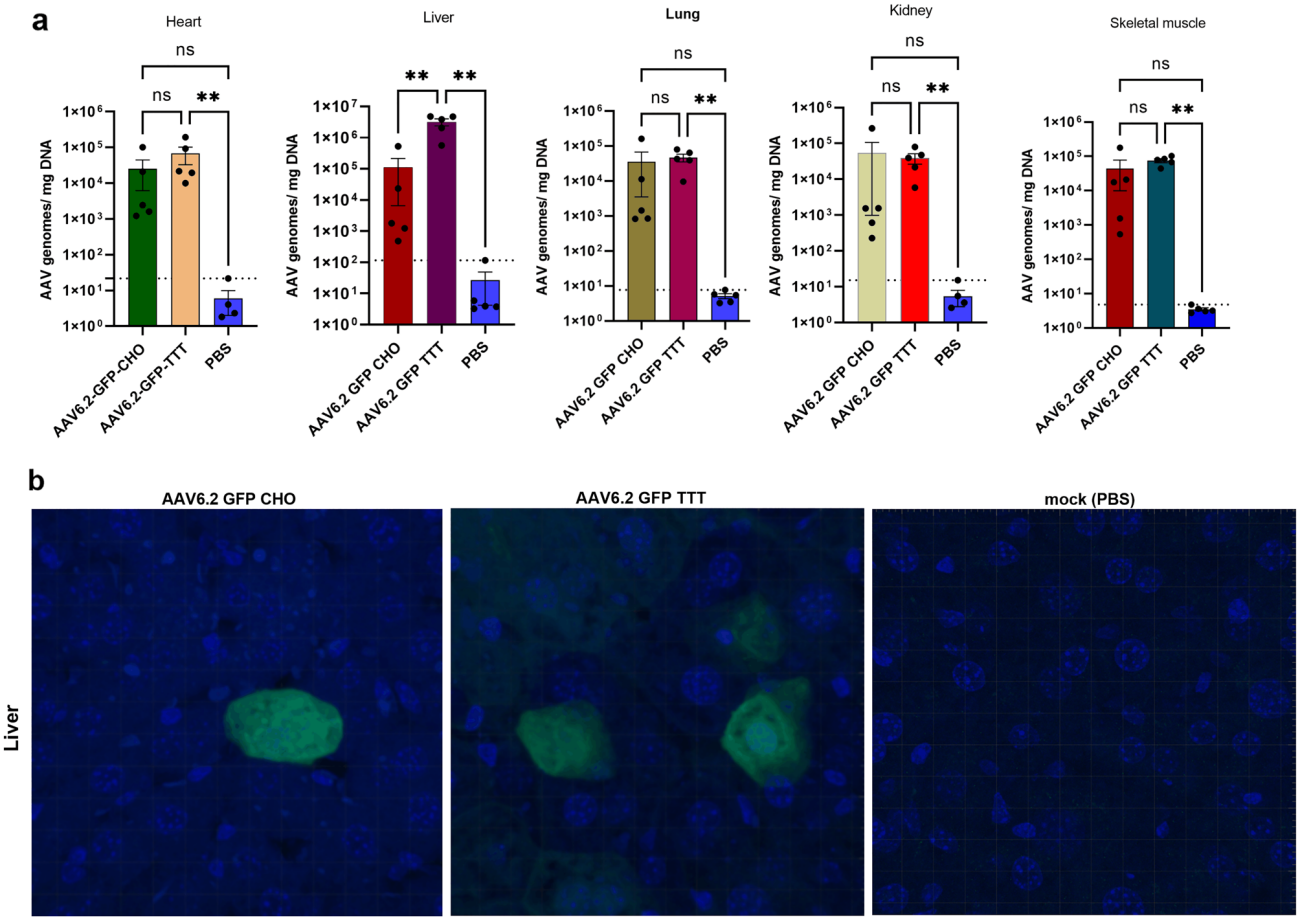
Biodistribution of rAAVs-derived from CHO cells in mouse tissues. (**a**) Genome copy numbers of rAAV6.2 GFP derived from CHO–HV–C1 or HEK293 isolated from heart, liver, and lung harvested tissues evaluated using qPCR.**p = 0.012. (**b**) Detection of GFP expression from harvested liver sections (G1, G2 and G3) using confocal microscopy. PBS was used as a negative control (mock).

Samples were assessed for rAAV titer in homogenized tissue using qPCR (targeting the GFP gene), and histopathologic examination was performed using confocal microscopy. From the qPCR analysis, mice from G1 showed lower rAAV6.2-GFP genome copy numbers than mice from G2 in all harvested tissue, without a significant statistical difference except for liver (*p* = 0.0012). (Fig. 7a). Thin liver tissue sections from the three groups were prepared and examined for GFP expression using confocal microscopy. Clear GFP signals were observed in the livers of all inoculated groups except G3 (Fig. 7b), as expected from the qPCR data. These data indicate that rAAV produced in engineered CHO cells show good biodistribution after tail vein injection. Additionally, the lower GFP in G1 vs. G2 liver sections may be due to the presence of impurities that cannot be eliminated with PEG-Chloroform method.

### Reengineering CHO–HV–C1 cells for HSV-1 production

**Figure 8:**
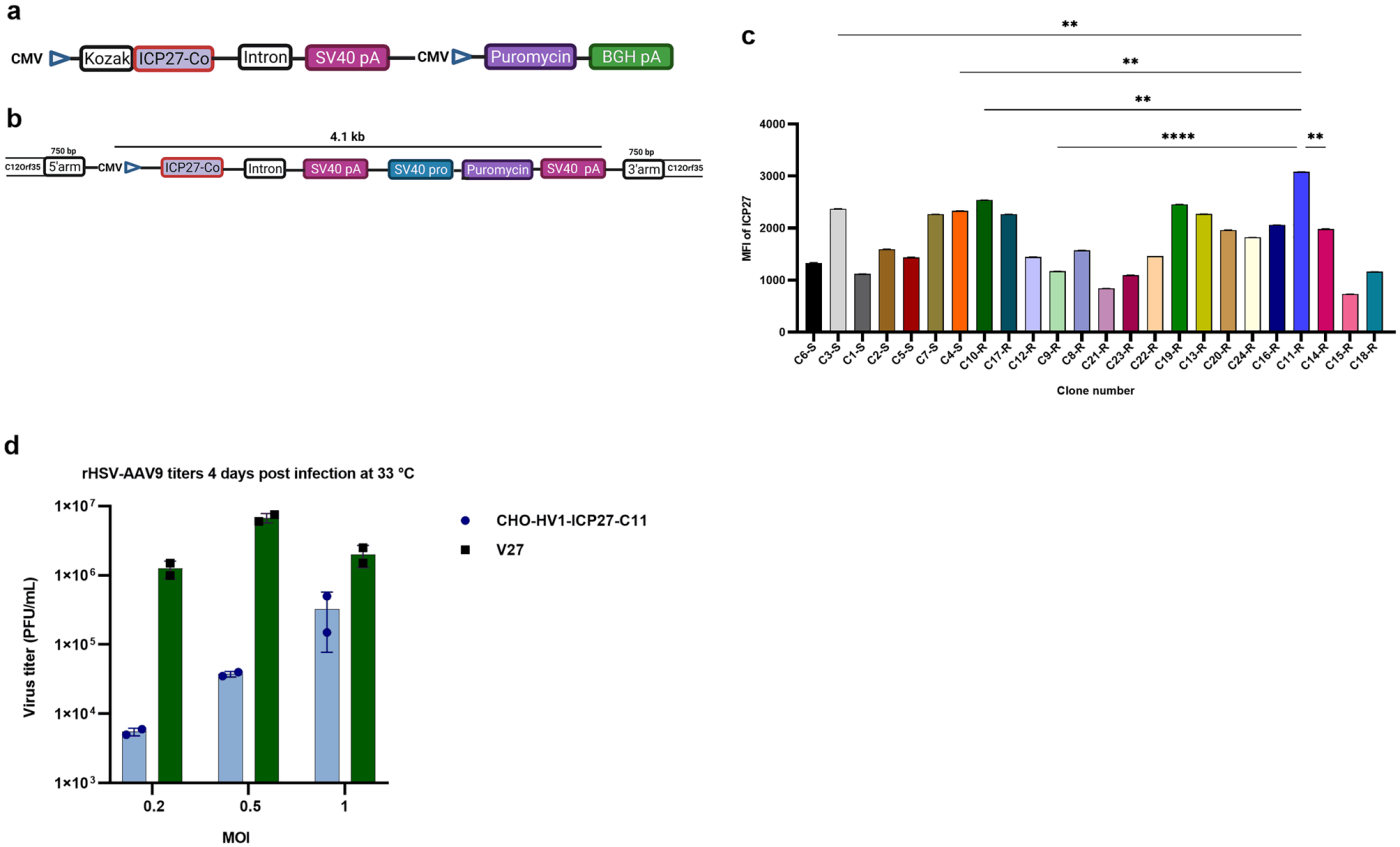
Development and selection of HSV-1 producer CHO-ICP27 pools and clones. (**a**) Construct for development of stable CHO-HV1-ICP27 pools using random integration. The Chinese hamster codon-optimized HSV-1 ICP27 ORF expression was driven by CMV promoter under puromycin selection. (**b**) Construct for development of stable CHO-HV1-ICP27 pools using CRISPR/Cas9 technology. Donor plasmid was constructed and encoded codon-optimized Chinese hamster ICP27 and puromycin cassettes flanked by two homology arms. (**c**) Mean fluorescence intensity (MFI) of ICP27 expression from the final selected clones. Data (n = 2 per sample) are expressed as mean ± SD. (**d**) Comparison of rHSV-AAV9 production in CHO-HV1-ICP27-C11 and V27 cells using different MOIs. Data were analyzed with two-way ANOVA and reported as mean ± SD.

In order to replace adherent, complimenting V27 cells for large-scale rHSV-1 production, we aimed to engineer our CHO–HV–C1 cells to express ICP27, an immediate early protein necessary for expression of late HSV-1 gene products. We utilized two strategies to generate HSV–1–ICP27 producer clones in our engineered CHO cells. The first strategy was random integration using one plasmid (Fig. 8a). The second strategy was site-specific integration using CRISPR/Cas9 with two plasmids: the first plasmid had a cassette composed of CHO codon-optimized HSV-1 ICP27 ORF downstream of the CMV promoter and upstream of the SV40 polyA, with a puromycin selection cassette; the second plasmid encoded synthetic sgRNA for CHO exon 1 C12orf35^31^. The total length of the two cassettes was 4.1 kb flanked by the right and the left homology arms (750 bp each) (Fig. 8b).

Pools from either random or site-specific integration transfected cells were recovered after three weeks of double selection using 5 µg/mL of puromycin and 50 µM MSX. Twenty-four clones showing high growth, viability, and ICP27 expression were selected, including seven site-edited clones (clones 1–7) and 17 random-integrated clones (clones 8–24) (Fig. 8c**)**. Random-integrated clone #11 (CHO-HV-ICP27-C11), which demonstrated the highest growth profile and ICP27 expression, was selected to be tested for rHSV-1 vector production.

The CHO-HV-ICP27-C11 clone was infected with rHSV-AAV9 (MOI = 10 PFU/cell) and incubated at 37 °C for 2 h for virus adsorption. After 2 h, infected cells were washed twice with sterile 1 × PBS to remove any viral residues. The infected cells were then incubated for 24 h at 37 °C in a 5% CO_2_ static humidified incubator. On the second day, 1 mL of clarified supernatant from infected cells was passaged on to the V27 cells. The appearance of cell rounding and detachment of the infected cell sheet appeared two days post-infection. Additionally, expression of HSV-1 glycoprotein D (gD) was observed in the V27 cell lysate after infection with the rHSV-1 virus propagated in clone CHO–HV–ICP27–C11 (data not shown). These results indicates that CHO–HV–ICP27–C11 clone supports productive infection for rHSV-1 vectors.

We aimed further to compare the production capability of CHO–HV–ICP27–C11 clone to the V27 cells. Different MOIs (0.2, 0.5 and 1 PFU/cell) of rHSV–AAV9 were used to infect either CHO–HV–ICP27–C11 or V27 cells, using either a serum-free in-house medium or DMEM supplemented with 2% (*v/v*) FBS (GE Hyclone), respectively. The infected cell cultures were incubated for 4–5 days at 33 °C, 5% CO_2_ in a humidified incubator. rHSV-AAV9 from infected cell cultures were released by three freeze–thaw cycles and titrated by plaque assay in V27 cells. On days 4 and 5 post-infection, CHO–HV–ICP27–C11 cells produced significantly lower rHSV-AAV9 titers of 5.0 × 10^3^, 4.0 × 10^4^ and 3.2 × 10^5^ PFU/mL at MOIs 0.2, 0.5 and 1 PFU/cell, respectively, compared to V27 cells that produced 1.0 × 10^6^, 6.8 × 10^6^ and 2.0 × 10^6^ PFU/mL at MOIs 0.2, 0.5 and 1 PFU/cell, respectively (Fig. 8d). This result indicates that CHO-HV-ICP27-C11 cells have a lower capability for production of rHSV-1 than V27 cells.

### Discussion

Gene therapy has great potential to address the lack of life-altering treatments or cures for many different diseases such as cystic fibrosis and heart failure. In recent years, gene therapy research has focused on the use of AAV vectors because of their long-lasting expression after delivery to target organs and their non-pathogenic profile in humans. Previous studies showed that rHSV-1-assisted rAAV production provides a highly efficient manufacturing method^3^. However, typical manufacturing of rAAV using the rHSV-1 system involves infection of virus production cells, such as BHK-21 or HEK293 cells, with two replication-deficient rHSV-1 vectors in a serum-supplemented medium. This approach is very expensive for large-scale production and may introduce adventitious viral agents and/or prions into the final product^46–51^. Another drawback of manufacturing rAAV using the rHSV-1 system is the challenge in generating sufficient rHSV-1 vector stock for clinical and commercial manufacturing, as the scale-up process currently depends on using adherent Vero cells expressing the HSV-1 ICP27 protein (V27 cells)^3^.

CHO cells are the predominant mammalian cell type used to produce recombinant protein biologics, due to their ability to correctly fold, assemble, and modify recombinant proteins^22,32,33^. Certain animal cell types, such as swine testis (ST) and CHO cells, can bind HSV-1 virus efficiently but restrict viral entry^34,35^. In addition, it has been reported that ST and CHO cells become susceptible to HSV-1 entry upon expression of human cDNA encoding HVEM^24^. Here, we aimed to engineer suspension, serum-free-adapted CHO cells to produce different rAAVs using the HSV-1 helper system, a virus which naturally does not infect wildtype CHO cells.

We have successfully engineered suspension, serum-free-adapted CHO cells to express HVEM and/or Nectin-1 proteins, which are necessary for rHSV-1 entry and infection. All of the engineered stable CHO pools showed significant susceptibility to rHSV-1-GFP vector entry and infection, as evidenced by GFP expression, compared to the wild-type CHO cells. Interestingly, the CHO cell pools expressing HVEM only outperformed all other pools in GFP expression when infected with the rHSV1-GFP vector. These data agree with the previous publication, which reported that engineered CHO cells expressing the HVEM receptor became permissive to HSV-1 entry and infection^24^. In addition, it has been reported that HVEM receptor supports more HSV-1 infection in CHO cells than Nectin-1^35^. Interestingly, the HVEM-only pool also outperformed all other stable pools in producing high rAAV9-GFP vector titers from the cell culture medium 24 hpi, which encouraged us to generate clonal cells from this pool.

Following optimization of the infection parameters, clone CHO–HV–C1 produced various AAV serotypes at titers higher than previously reported in CHO cells (~ 10^10^ vg/mL at 24 hpi). A MOI of 4:1 was optimum for production of rAAV6.2, rAAV8 and rAAV9 expressing GFP, with no additional improvement seen at higher MOIs for rAAV6.2 (data not shown). Additionally, shifting temperature from 37 to 33 °C enhanced release of rAAVs into the medium, particularly for rAAV8 and rAAV9. These data are very promising, as the CHO-HSV-1 platform can produce high rAAV titers with a lower MOI than that required for BHK-21, where a MOI of 12:2 was reported as optimal^3^. The lower MOI for the CHO-HSV-1 platform will benefit the biomanufacturing capability, particularly for the yield and purification of HSV-1 from rAAVs.

It should also be noted that a sharp decrease in the CHO–HV–C1 cell viability post co-infection at 37 °C was observed during production of different AAV serotypes. However, this decrease was minimized by switching the infection temperature to 33 °C. It has been reported that the stability of rHSV-1 vectors was 2.5-fold greater at 33 °C than 37 °C, and rHSV-1 synchronous infection incubated at 33 °C produced twofold higher amounts of vectors than those incubated at 37°C^36^. Additionally, the Rep protein encoded in the rHSV-1 vector may exert a toxic effect on the infected CHO–HV–C1 cells after infection at 37 °C; however, this point needs further investigation.

Using the PEG-Chloroform purification method followed by concentration using the 100 kDa molecular-weight cutoff (MWCO) sample concentrator, we were able to purify rAAVs produced in our engineered CHO cells in one day. The developed purification method is simple, cheap, and fast, with titers comparable with the more time-consuming iodixanol ultracentrifugation method, which agrees with other studies^27–29^. Moreover, no difference in either in vitro or in vivo transduction of rAAV purified with Chloroform/PEG or iodixanol ultracentrifugation was observed^37^. Additionally, rAAVs produced in our engineered CHO cells and purified with the PEG-Chloroform method showed potent in vitro transduction and infectivity comparable to those produced using the triple transient transfection^38^ in HEK293 cells and purified with column chromatography^39^.

The AAV capsid gene produces viral proteins, VP1, VP2, and VP3, that assemble to form the *T* = 1 icosahedral capsid, consisting of 60 total VPs. The VP composition of the AAV capsid is estimated to be at a molar ratio of 1:1:10^7,40,41^, such that one AAV capsid likely has 5 VP1, 5 VP2, and 50 VP3. Interestingly, analysis of the composition of the capsid protein ratio of rAAV6.2-GFP and rAAV9-GFP vectors produced in the CHO–HV–C1 cells using CE-SDS showed very comparable VP1:VP2:VP3 capsid ratios to those reported in the literature (data not shown). This result indicates that rAAVs produced in CHO–HV–C1 cells are functionally assembled, in contrast to the insect cell-baculovirus platform where genetic engineering and/or process optimization is required to enhance VP expression of some AAV serotypes. For example, early trials to adapt AAV5 for production using the insect cells-baculovirus platform showed low levels of VP1 incorporation into the capsid^52^ that was improved later^53–55^. Examination of full and empty capsids for rAAVs produced in the CHO–HV–C1 clone showed higher full capsid percentage than what has been reported previously from an optimized rHSV-1 co-infection in BHK-21 cells^3^. However, the use of PEG in our protocol may have an impact on increasing the full capsid percentage, and future purification experiments using chromatography are necessary.

We thought engineering CHO–HV–C1 cells to produce rHSV-1 stock would simplify the whole (AAV and rHSV-1) manufacturing process. Therefore, to address the current challenge in scaling-up rHSV-1 vector stock, we engineered CHO–HV–C1 cells to express the rHSV-1 ICP27 protein. Using an in-house medium, the top clone, CHO–HV1–ICP27–C11, showed productive infection for rHSV-1, as indicated by replication of CHO-derived rHSV-1 on V27 cells. However, the production capacity of rHSV-1 vectors from CHO-HV1-ICP27-C11 was lower compared to V27 cells. Our preliminary expression profiles show that expression of some late HSV-1 viral proteins in the infected CHO-HV1-ICP27-C11 cells were lower than expression of early and intermediate viral genes. Additionally, CHO cells may not provide the elements necessary for the optimal expression of many HSV genes, particularly late genes, because CHO cells may express some inhibitory factors that interfere or block HSV-1 late viral genes expression. These findings agree with a previous study^24^. However, further investigation is necessary to understand if factors related to the in-house medium can potentially have some inhibitory effect for rHSV-1 vector production.

### Concluding remarks

In summary, the engineered CHO–HV–C1 cells can produce multiple AAV serotypes at satisfactory titers using a serum-free medium with a lower MOI for HSV-1 vectors, compared to other studies that used a high MOI for HSV-1 vectors for AAV production in BHK-21 and HEK293 cells. We have observed that rHSV-1 MOI and incubation temperature after rHSV-1 co-infection are critical factors for production of a high rAAV titer in the CHO system. Moreover, low incubation temperature may have an impact on lowering the toxic effect of the *rep* gene on the producer cells. The optimum harvesting time for rAAV produced in the CHO cells was only 24 h post co-infection, which indicates the production and purification processes could be completed in as little as 2–3 days. The rAAVs produced in our engineered CHO cells showed high full capsid percentage and good capsid protein expression, transduction, and infectivity in vitro, with good biodistribution. There are some limitations in our study, including the need for optimization of purifying rAAV produced in CHO cells using chromatographic methods. Taken together, the HSV-based rAAV production platform in the engineered CHO cells provides a novel, scalable, serum-free manufacturing platform that will facilitate the production of future rAAV-based biotherapeutics in a low-cost manner.

## Materials and methods

### Generation of stable CHO pools

The open reading frames (ORFs) of human HVEM and Nectin-1 were downloaded from the NCBI database (accessions U70321.1 and AF060231.1, respectively). Both HVEM and Nectin-1 ORFs were codon-optimized for expression in hamster cells using online tools (https://www.idtdna.com/CodonOpt [IDT] and https://www.thermofisher.com/us/en/home/life-science/cloning/gene-synthesis/geneart-gene-synthesis/geneoptimizer.html [Thermo Fisher Scientific, CA, USA], respectively). The HVEM was codon-optimized by IDT’s tool using their property in-house algorithm with the default setting and human as the target species for the expression. The Nectin-1 was codon-optimized using GeneArt’s tool using their default setting and human as the target species for expression as well. The delivered plasmids encoding synthetic HVEM or Nectin-1 were sub-cloned into an in-house plasmid downstream of the enhanced human cytomegalovirus (CMV) promoter or a synthetic promoter^42^, generating eight different constructs. All constructed plasmids encoded glutamine synthetase (GS) under the control of the SV40 promoter allowing for the selection of transfected cells in methionine sulfoximine (MSX). All final plasmids were verified by whole plasmid sequencing (Psomagen, MD, USA). Proprietary in-house, suspension, serum-free-adapted CHO host cells were cultured in CD-CHO medium (10,743,029; Gibco, MD, USA), supplemented with 6 mM L-glutamine (part number 35050–06; Gibco, MD, USA), dextran sulphate (49,110; Sigma-Aldrich, MA, USA) and incubated in a 6% CO_2_ humidified incubator at 37 °C and 120 rpm agitation. Viable cell density (VCD) and percent viability were measured daily using a Vi-Cell XR automated cell counter (Beckman Coulter, IN, USA).

Stably-transfected CHO pools were generated following an in-house protocol. In brief, eight aliquots of CHO cells (1 × 10^7^ viable cells per aliquot) were pelleted for 5 min at 200 × g. Cell pellets were mixed with 7 µg of each purified linearized in-house plasmid and then transfected using the Amaxa cell line nucleofector Kit V (VCA-1003; Lonza, MA, USA), according to the manufacturer’s instruction. After 24 h post-transfection, cell viability was measured, and MSX (M5379-1G; Sigma–Aldrich, MA, USA) was added to each pool for recombinant cell selection.

Aliquots (1 × 10^6^ viable cells per pool) from recovered cell pools were tested for HVEM and Nectin-1 receptor expression using flow cytometry. Briefly, cell pools were incubated with either 150 µL of 1:200 diluted Anti-HU CD270-PE (clone eBioHVEM-122, 12–5969-80; Invitrogen, MA, USA) or Nectin-1-PE (clone R1.302-PE, Ma5-28,593; Invitrogen, MA, USA) antibodies for 15 min at room temperature in the dark, for pools expressing HVEM and Nectin-1, respectively. All antibodies were diluted in D-PBS (D8537; Sigma-Aldrich, MA, USA) supplemented with 1% bovine serum albumin (BSA, P/N 56,773; Invitrogen, MA, USA). After incubation, cells were pelleted by centrifugation, washed twice with sterile D-PBS, and then fixed with Fix and Perm Medium A (GAS002S100; Life Technologies, MA, USA) for 15 min at room temperature in the dark. The stained cells were washed twice with D-PBS and resuspended in flow staining buffer (D-PBS supplemented with 0.1% BSA), followed by analysis in LSR II cytometer (BD Biosciences, CA, USA). Data analysis was performed using FlowJo v10.0 software (Tree Star, Inc, CA, USA).

### rHSV-1-GFP infection in stable CHO pools

The rHSV1-GFP was constructed by cloning the GFP flanked by AAV2 ITRs into HSV-1 DNA using standard molecular cloning techniques. All rHSV-1 vectors have been generated in V27 cells using homologous recombination. An aliquot (1 × 10^6^ viable cells) from each recovered cell pool, as well as the CHO host cells, were infected with rHSV1-GFP (MOI = 10 PFU/cell) in a 6-well cell culture plate (3516; Corning, NY, USA) and incubated for 1 h at 37 °C in a humidified 5% CO_2_ incubator. After one hour, infected cell pools were centrifuged for 5 min at 1200 rpm, and the virus supernatant was discarded. Infected cell pellets were resuspended into CD-CHO medium and incubated at 37 °C in a 5% CO_2_ humidified incubator. Cells from each of the infected pools were imaged every 12 h for GFP expression using IncuCyte (Sartorius, Germany) with its default settings, for a total five time points.

### rAAV9-GFP vector production in stable CHO pools

An aliquot of each cell pool (1 × 10^6^ viable cells) was infected with MOI (1:1 PFU/cell) from rHSV-AAV9 *rep/cap*:rHSV1-GFP vectors into 6-well culture plates (Corning, NY, USA). Infected cell pools were incubated for 24 h at 37 °C in a 5% CO_2_ humidified incubator. After 24 h, the infected cell supernatant was collected and tested for rAAV9-GFP titer using qPCR. Collected supernatant was digested at 37 °C for one hour with DNase I (200 U/µL, 18,047,019; Invitrogen, MA, USA) followed by a 10-min incubation at 95 °C for enzyme inactivation. The digested samples were then incubated with an equal volume of proteinase K (100,005,393; Invitrogen, MA, USA) digestion mix (200 mM NaCl, 20 mM Tris–HCl, pH 8.0, 2 mM ethylenediaminetetraacetic acid, pH 8.0; 0.5% sodium dodecyl sulfate, proteinase K (20 mg/mL) at 55 °C for one hour followed by enzyme inactivation for 10 min at 95 °C. The reaction for absolute qPCR quantification was done using a PCR thermocycler (QuantaBio Q, Qiagen, MA, USA) with an in-house standard linearized AAV plasmid in a 20 µL reaction containing TaqMan Fast Universal PCR 2X Master Mix (4,352,042; Applied Biosystems, MA, USA) and 20 µM each of CMV-Forward primer (5′-TTCCTACTTGGCAGTACATCTACG-3′), CMV-Reverse primer (5′-GTCAATGGGGTGGAGACTTGG-3′), and CMV probe (5′-FAM-TGAGTCAAACCGCTATCCACGCCCA-NFQ-3′), in addition to 5 µL of diluted template. The PCR cycling profile was 95 °C for 2 min and 40 cycles of 95 °C for 5 s and 60 °C for 30 s.

### Generation of CHO-HVEM expressing clones and testing of rHSV1-GFP infection

Single‐cell deposition was performed using a BD Influx cell sorter (BD Biosciences, CA, USA) according to Evans et al., 2015. In brief, an aliquot containing 3 × 10^6^ viable CHO HVEM expressing cells were stained with 1:200 anti-human CD270 (HVEM)-PE antibody for 15 min in the dark at room temperature. Stained cells were washed twice with sterile PBS (RNBK5430; Sigma-Aldrich, MA, USA), pelleted at 200 × g for 5 min, and then resuspended in 1 mL sterile PBS. Single cells from the PE‐gated fraction were deposited into each well of two 384‐well plates (4588; Corning, NY, USA) containing in-house conditioned medium supplemented with MSX. The clonal cell lines were selected for testing for rHSV1-GFP infection, and the mean GFP expression was calculated from two time points (24 and 48 h post-infection) using IncuCyte as previously described.

### Production of rAAV6.2-GFP vector in selected CHO-HVEM clones

The selected clones were further tested for rAAV6.2-GFP vector production using different MOIs for rHSV-AAV6.2 *rep/cap*:rHSV-GFP. All infected clones were incubated at 37 °C in a 5% CO_2_ humidified incubator with 120 rpm agitation for three days. An aliquot (1 mL) from each co-infected clone was harvested at 24 and 48 h post co-infection and centrifuged at 200 × g for 5 min. The harvested samples (cell pellets) were then prepared for rAAV titration using qPCR. In brief, infected cell pellets were collected, mixed with the AAV lysis buffer (50 mM Tris, pH 8.0, 150 mM NaCl), and subjected to three cycles of freeze/thawing in an isopropanol dry ice bath followed by centrifugation at 12,000 rpm for 30 min at 4 °C. After centrifugation, the supernatant was collected and the rAAV6.2-GFP titers were determined using qPCR, as previously described.

### Purification and analytical characterization of CHO-derived rAAVs

The rAAVs produced in the top CHO clone were purified using the polyethylene glycol (PEG; BP2331; Fisher Scientific, MA, USA) chloroform method^27^ with some modifications, including using 0.5% Triton X-100 (CLCF6075; Sigma-Aldrich, MA, USA) for cell lysis and rHSV-1 inactivation, followed by precipitation using 8% polyethylene glycol 8000 and 150 mM NaCl (S9888-1 kg; Sigma-Aldrich, MA, USA). Viral pellets were further treated with Benzonase (E1014-5KU; MilliporeSigma; 50 U/mL, MD, USA) and RNase (AM2270; Invitrogen; 10 µg/mL) at 37 °C for 1 h followed by 1:1 chloroform (C2432-1L; Sigma-Aldrich, MA, USA) treatment. The aqueous layer post chloroform treatment was collected and concentrated using AMICON filters (UFC500324; Millipore, MD, USA). The concentrated viruses were stored at − 80 °C until further use.

Purified rAAVs were tested for capsid protein expression using Western blotting. In brief, purified rAAV6.2-GFP and rAAV9-GFP vectors produced in the top clone using different MOIs of rHSV-1 vectors were prepared for SDS-PAGE gel by adding the appropriate volume of 4X NuPAGE lithium dodecyl sulfate (LDS) sample buffer (NP0007; Invitrogen, MA, USA) and 10X NuPAGE sample-reducing agent (NP0004; Invitrogen, MA, USA). The samples were then incubated at 70 °C for 10 min. Equal volumes (20 µL) of rAAVs were loaded on a Bolt 4–12% Bis–Tris plus 12-well gel (Invitrogen, MA, USA) for each serotype and were run using 1X NuPAGE running buffer (NP0001; Invitrogen, MA, USA). After the run, the gel was subjected to dry transfer using an iBolt 2NC mini stack (IB301002; Invitrogen, MA, USA) followed by blocking in 5% skimmed milk (M203-10G; Amresco, MA, USA) diluted in TBS (1,706,435; Bio-Rad, MA, USA) for one hour at room temperature. After blocking, the membrane was incubated with AAV VP1, VP2, VP3 antibody (GTX44495; GeneTex, CA, USA) diluted 1:200 with 5% skimmed milk overnight at 4 °C with gentle shaking. After incubation, the blotted membrane was washed three times with TBS supplemented with 0.1% Tween (003,005; Life Technologies, MA, USA) and then incubated with 1:100 goat anti-mouse IgG (SSA006; Sino Biological, TX, USA) for 1 h followed by washing. The membrane was incubated with Supersignal West Pico Plus Substrate (34,580; ThermoFisher Scientific, MD, USA) before image detection using Amersham Imager 680 (GE Healthcare, UK).

Purified rAAVs from the top CHO clone were visualized using mini-transmission electron microscopy (Mini-TEM; Vironova, Sweden). In brief, PEG-Chloroform purified rAAV6.2-GFP and rAAV9-GFP samples were placed on a 400-mesh glow-discharged carbon grid by first inverting the grid and placing it on top of a 10 μL droplet of rAAV, deposited on parafilm, for 30 s. Excess sample was blotted off by gently touching the edge of the grid against a Whatman filter paper. The grid was then washed twice with two 20 μL droplets of double distilled water. The sample-containing grid was then stained with a 20 μL droplet of 1.5% uranyl acetate for 10 s. Excess stain was blotted off by gently touching the edge of the grid against a Whatman filter paper. The rAAV samples were then visualized using a Mini-TEM instrument.

The rAAVs capsid protein (VP1:VP2:VP3) ratio analysis was performed using an in-house developed capillary electrophoresis sodium dodecyl sulfate (CE-SDS) method^43^.

### Detection of residual infectious rHSV-1 from purified CHO-derived rAAVs

V27 cells expressing a stable copy of the rHSV-1 ICP27 protein were seeded at 0.5 × 10^6^ viable cells/well in a 6-well culture plate containing DMEM (11,995–065; Gibco, MD, USA) supplemented with 10% FBS and 500 µg/mL geneticin (10,131–035; Gibco, MD, USA) overnight. After 16 h, the cells were washed twice with sterile PBS and then infected with purified rAAV6.2-GFP vectors at a 1:100 dilution (~ 10^10^ vg/mL). The rHSV1-GFP vector was used as positive control at MOI = 0.15 PFU/cell. Infected cells were incubated for 2 h at 37 °C for virus adsorption. After incubation, the excess virus was removed, and the infection medium (DMEM supplemented with 2% FBS) was added. The plate was incubated and monitored with IncuCyte for 4 days to capture any cytopathic effects.

### In vitro infectivity of CHO-derived rAAVs

For infectivity analysis, Ad293 cells (240,085; Agilent, CA, USA) were seeded in a 96-well culture plate (2 × 10^4^ cells/well) and incubated at 37 °C in a humidified, 5% CO_2_ incubator overnight. Ten-fold dilutions of rAAV6.2-GFP produced in either engineered CHO cells or HEK293 cells expressing adenovirus E1a and E1b proteins. rAAV6.2-GFP produced in HEK293 cells using triple transfection. were prepared in 1X DMEM. Each virus dilution was used to infect four wells, and infected cells were incubated at 37 °C, 5% CO_2_ for 5 days. On day 5 post-infection, infected cells were imaged for GFP expression using IncuCyte (default settings), and virus titers were calculated^44^ and expressed as tissue culture infective dose 50 (TCID_50_). For transduction analysis, Ad293 cells were cultured as mentioned above, and then infected with different MOI from CHO-derived rAAVs, or similar vectors produced by in HEK293 cells using the triple transient transfection method, as described above.

### Biodistribution of CHO-derived rAAVs

All animal experiments were approved by the Institutional Animal Care and Use Committee of AstraZeneca (Gaithersburg, MD, USA). All animal experiments were performed in accordance with relevant guidelines and regulations. In addition, our study is reported in accordance with the ARRIVE guidelines.

Eight-week-old male C57bl/6 mice were purchased from Jackson Laboratory (Bar Harbor, ME, USA). Mice were divided into three groups (n = 5 per group), and all mice were inoculated with 1 × 10^11^ vg/100 µL of the appropriate rAAV vector (or saline) via the tail vein (TV) using an insulin syringe (Becton Dickinson, DC, USA). The inoculated mice were monitored daily for any clinical symptoms of illness. Three weeks post-injection, mice were euthanized via CO_2_ inhalation and organs were harvested. Half of the harvested tissues were frozen in dry ice in a microcentrifuge tube for qPCR analysis, and the other half were fixed in 10% neutral buffered formalin for histology work. DNA extraction from the harvested tissues was performed using the All-Prep DNA/RNA Mini Kit (80,004; Qiagen, MD, USA), according to the manufacturer’s instructions. The qPCR reactions were carried out on the QuantStudio 7 Flex (ThermoFisher Scientific, MA, USA) using in-house linearized plasmids, pAAV-GFP. The extracted genomic DNA (100 ng) was used as a template, with either specific GFP primers and probes (forward 5′-GAACCGCATCGAGCTGAA-3′, Reverse 5′-TGCTTGTCGGCCATGATATAG-3′, and probe 5′/56-FAM/ATCGACTTC/ZEN/AAGGAGGACGGCAAC/3IABkFQ 3′. Cycling conditions were initial denaturation at 95 °C for 30 s, 40 cycles of 95 °C for 10 s, and annealing/extension at 60 °C for 20 s. For histology, thin liver tissue sections from the collected tissues were prepared using a Leica 3050 s microtome (Leica, Germany), stained, mounted, and assessed for GFP expression using confocal microscopy.

### Engineering the CHO rHSV-1 vector producer cell line

Two strategies were used: (1) random integration, where HSV-1 ICP27 ORF (accession: AB235845.1) sequence was codon-optimized for hamster cells expression by IDT, chemically-synthesized, and then sub-cloned downstream the CMV promoter into an in-house plasmid encoding the puromycin cassette for selection; and (2) site-specific integration using CRISPR/Cas9 technology in which the exon1 from the C12Orf35 locus from CHO genome (accession: XM_027430029) was selected as one of the transcription hotspots^31^. The CRISPy bioinformatic tool^45^ with the default parameters was used to select the sgRNA target sequence. The selected sgRNA target (5′ GGACTTAACCACTCGATGGC 3′) was synthesized by IDT (IA, USA) and delivered as gblocks; it was annealed, and sub-cloned into the linearized CRISPR nuclease expression vector backbone to generate sgRNA expression vectors using the GeneArt CRISPR CD4 kit (A21175; Invitrogen, MA, USA), according to the manufacturer’s instructions. The donor DNA plasmid was constructed using the backbone of an in-house plasmid, avoiding any protospacer adjacent motif (PAM) sites identical to the gRNA target. The 5′ and 3′ homology arms (750 base pairs each) flanking the sgRNA target sequence were chemically synthesized by IDT. Transfection of the final AAV CHO clone was carried out using Amaxa nucleofector kit V as previously described. CHO cell pools expressing the ICP27 protein were selected using 5 µg puromycin/ml for two weeks. Clonal cell lines were generated as described above by single cell deposition in 384-well plates using anti-HU CD270 (HVEM)-PE antibody (clone eBioHVEM-122, eBioscience).

### rHSV-1 infection in final selected CHO-ICP27 clone

The final selected clone was tested for rHSV-1 infection. In brief, an aliquot (1 × 10^6^ viable cells) was infected with rHSV-AAV9 (MOI = 10 PFU/cell) in antibiotic-free DMEM, and then incubated for 20 min on ice, followed by incubation at 37 °C for 2 h. After incubation, the excess virus was decanted, and the infected cells were washed twice with sterile PBS to remove any residual rHSV-AAV9 vectors. After washing, infected cells were covered with an in-house medium (2 mL/well) in 6-well plate and incubated at 37 °C in a 5% CO_2_ humidified incubator for 24 h. After 24 h, 1 mL supernatant from the infected cells was collected and inoculated on V27 cells to determine cytopathic effect. Harvested viruses from the inoculated V27 cells were purified by ultracentrifugation and subjected to Western blot using anti-HSV-1 glycoprotein D antibody (10,418; Santa Cruz Biotechnology, TX, USA). The final CHO clone and V27 cells were infected with different MOIs of rHSV-AAV9 using the same approach described above. Infected cells were then incubated in 6-well plate containing an in-house medium (for CHO) or DMEM-2% FBS (for V27) for two days at 33 °C in a 5% CO_2_ humidified incubator. Clarified viruses from lysed cultures were harvested and titrated on V27 cells by plaque assay^3^.

### Statistical analysis

One-way ANOVA with Tukey–Kramer post-hoc analysis was used to compare GFP expression in CHO pools post rHSV-1 infection and to compare rAAV9-GFP titers produced in different CHO pools. Two-way ANOVA was used to compare rAAV6.2- GFP titers produced in different clones at two different time points. A two-tailed Mann–Whitney test was used to compare infectivity titers of rAAV6.2-GFP produced in CHO and HEK293 cells produced. The Kruskal–Wallis test with Dunn’s test for correction was used to compare rAAV6.2-GFP qPCR titers among the harvested mice tissues. GraphPad Prism version 9.1.2 (GraphPad Software Inc, CA, USA) was used for all tests and a *p-value* of ≤ 0.05 was considered a significant difference.

### Supplementary Information

Supplementary Figures.

## Data Availability

The authors confirm that the datasets generated and/or analyzed during the current study are available within the article and its supplementary information.
