# Supplementary material for: Engineered CHO cells as a novel AAV production platform for gene therapy delivery
Source: Sci Rep. 2023 Nov 6;13:19210. doi: 10.1038/s41598-023-46298-3 (PMC10628118; doi:10.1038/s41598-023-46298-3)
Supplement: Supplementary file 1 — Supplementary Figures. [file 41598_2023_46298_MOESM1_ESM.docx]

**Engineered CHO cells as a novel AAV production platform for gene therapy delivery**

Abdou Nagy^1*^, Lina Chakrabarti^1^, James Kurasawa^2^, Sri Hari Raju Mulagapati^3^, Paul Devine^4^, Jamy Therres^1^, Zhongying Chen^5^, Albert E Schmelzer^1*^

^1^ Cell Culture and Fermentation Sciences, Biopharmaceutical Development, BioPharmaceuticals R&D, AstraZeneca, One MedImmune Way, Gaithersburg, MD 20878, USA.

^2^ Biologics Engineering, R&D, AstraZeneca, One MedImmune Way, Gaithersburg, MD 20878, USA.

^3^ Analytical Science, Biopharmaceutical Development, Biopharma R&D, AstraZeneca, One MedImmune Way, Gaithersburg, MD 20878, USA.

^4^ Analytical Science, Biopharmaceutical Development, Biopharma R&D, AstraZeneca, Milstein Building, Granta Park, Cambridge, CB216GH, UK.

^5^ Clinical Pharmacology and Safety Sciences, AstraZeneca, One MedImmune Way, Gaithersburg, MD 20878, USA.


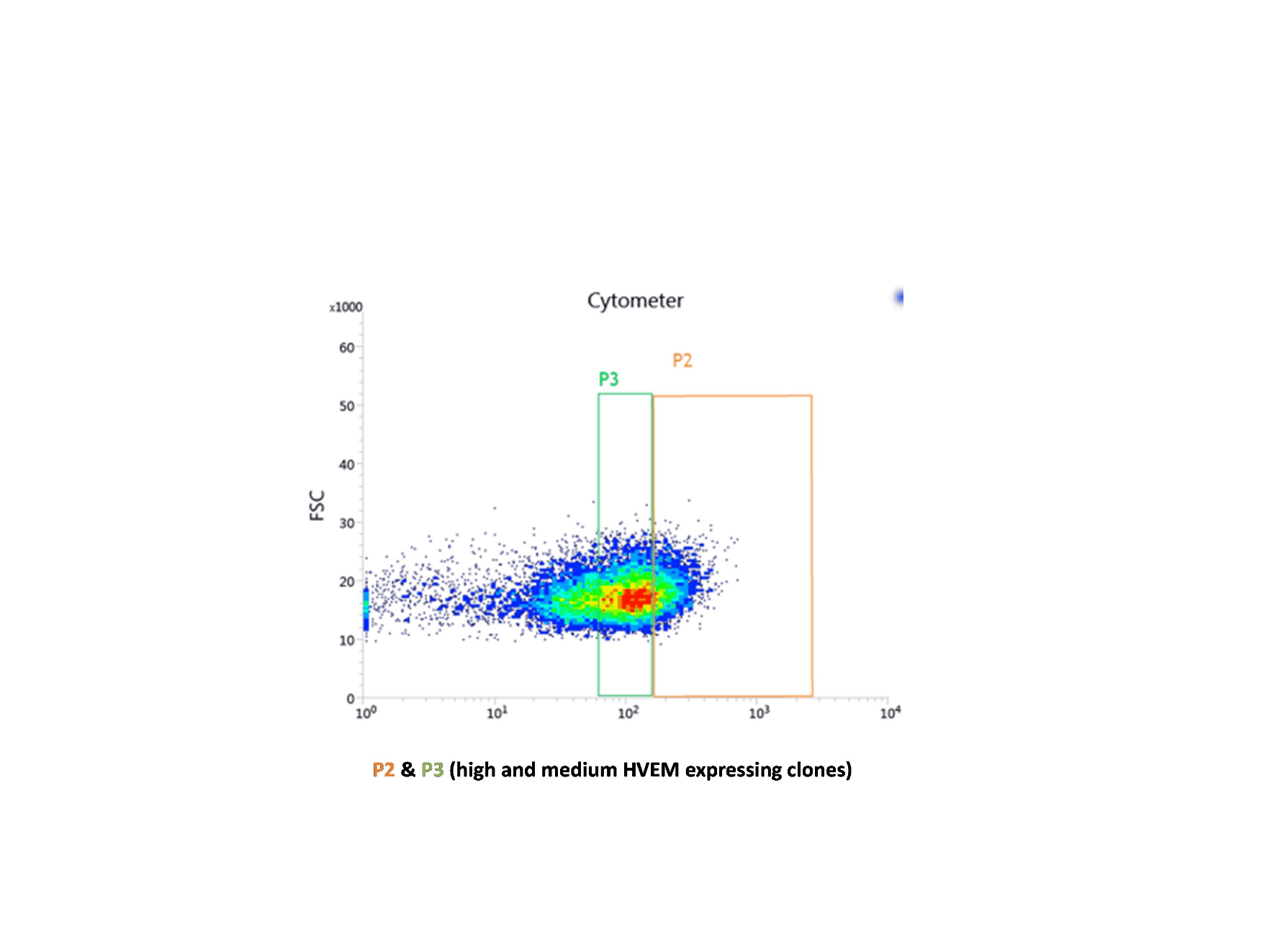
**Figure S1.** Flow cytometry gating strategy. High and medium HVEM-expressing clones were identified and selected for single cell deposition (P2 and P3, respectively).

**Figure S2.** Temperature switch post rHSV-1 co-infection. (a) Effect of temperature switch on cell viability post co-infection. (b) Effect of temperature switch on rAAV6.2 titers in cell culture medium and cell lysate.


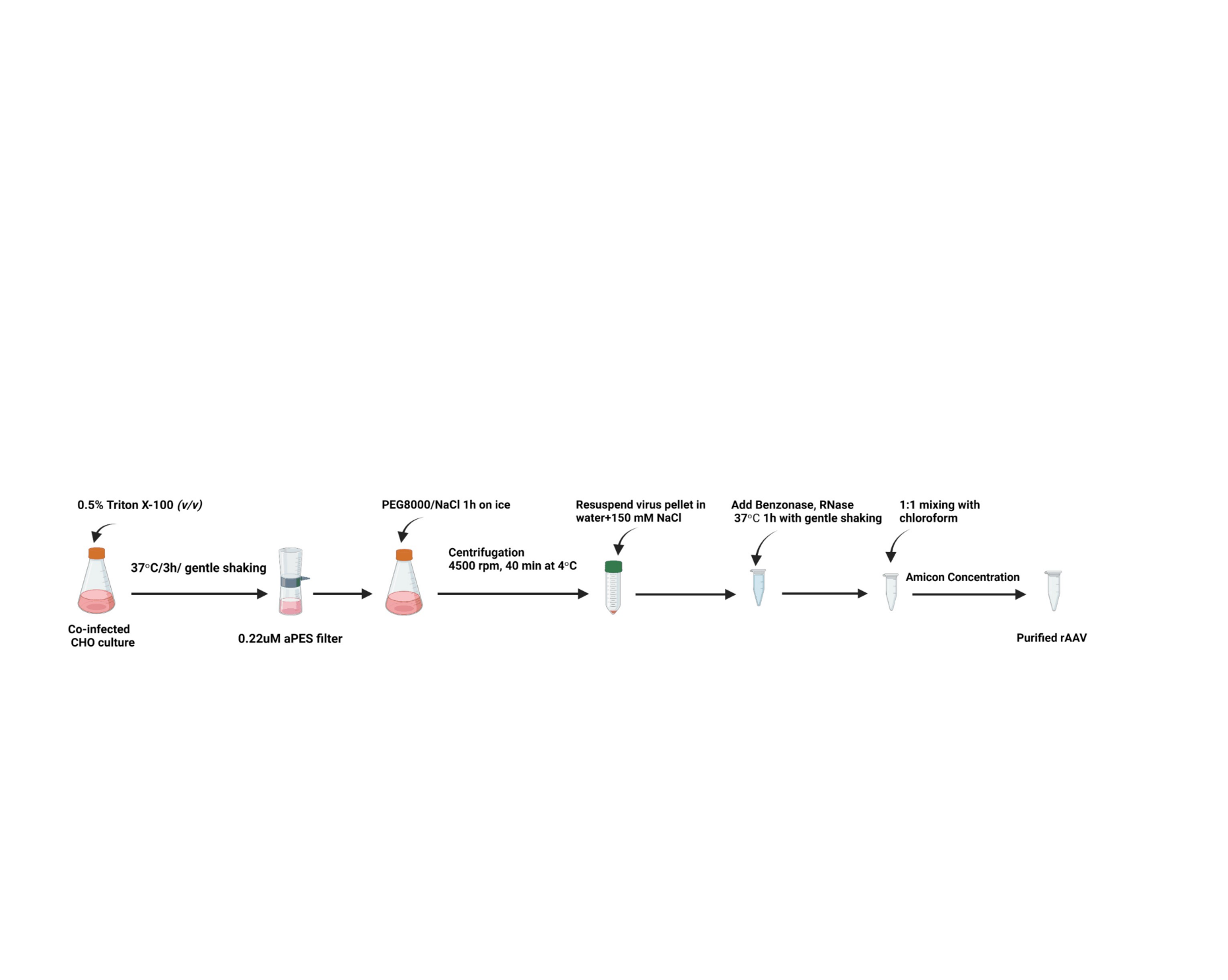


**Figure S3.** Workflow of the PEG-Chloroform rAAV purification method used for CHO cells. The figure was generated with BioRender.com


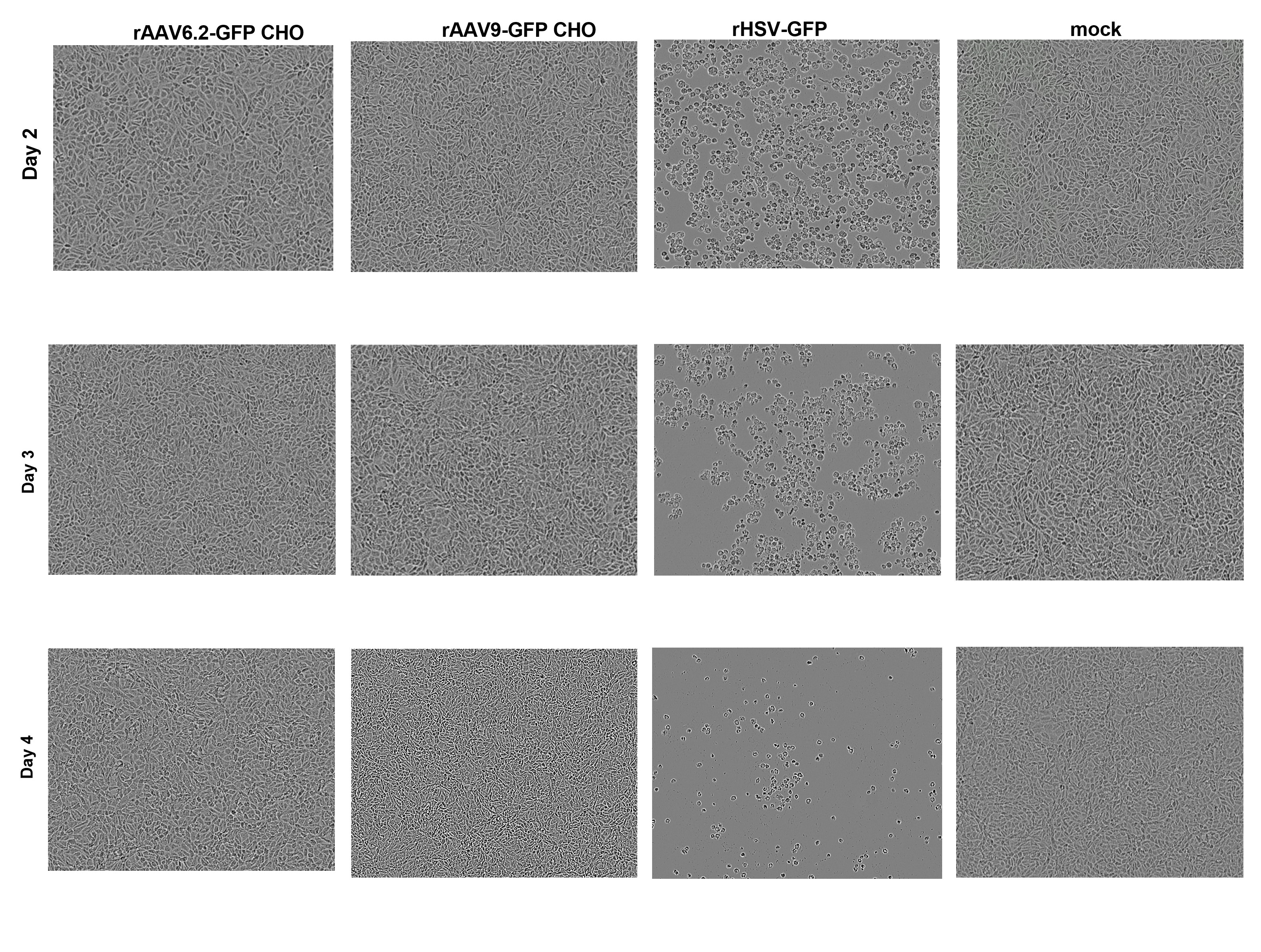


**Figure S4.** Evaluation of infectious rHSV-1 residues in purified rAAVs on V27 cells. Complementing V27 cells that stably express HSV-1 ICP27 protein were tested for any infectious residues of rHSV-1 vectors used in production of rAAVs in CHO-HV-C1 clone. PBS was used as a mock infection control.
